# Impact of Diabetes Mellitus On In-Patient Mortality From Pneumonia Over 25 Years in a Portuguese Tertiary Care Hospital

**DOI:** 10.7759/cureus.99912

**Published:** 2025-12-23

**Authors:** João Menino, Juliana Gonçalves, Mariana Azevedo, Jorge Pedro, Beatriz Prista-Leão, João Sergio Neves, Marta Borges-Canha, Joana Queirós

**Affiliations:** 1 Endocrinology, Diabetes, and Metabolism, Unidade Local de Saúde de São João, Porto, PRT; 2 Medicine, Faculdade de Medicina da Universidade do Porto, Porto, PRT; 3 Infectious Diseases, Unidade Local de Saúde de São João, Porto, PRT; 4 Surgery and Physiology, Faculdade de Medicina da Universidade do Porto, Porto, PRT

**Keywords:** diabetes mellitus, hospital mortality, pneumonia, retrospective studies, vaccination

## Abstract

Introduction

Pneumonia is among the most important causes of morbidity and mortality in Portugal. Diabetes mellitus (DM) is a risk factor for the development of pneumonia and has an adverse effect on the patients’ outcome. This study aimed to evaluate the evolution of in-hospital mortality from pneumonia in patients with DM over 25 years.

Materials and methods

Hospital admissions due to pneumonia were retrospectively assessed between 1989 and 2014 in patients with and without DM. Evolution of the annual number of admissions, length of stay, and in-hospital mortality was evaluated.

Results

A total of 1,356,502 hospitalizations were evaluated, of which 44,301 were due to pneumonia (20.3% had DM). The proportion of hospitalizations due to pneumonia was higher among patients with DM (7.2% vs 2.9%, p < 0.001). In this group, the proportion of female patients was higher (46.7% vs 41.3%, p < 0.001). Patients with a diagnosis of DM had a longer hospital stay (11 (7-20) vs 10 (6-19)). In-hospital mortality was higher among patients with DM (p < 0.001) across the study periods up to 2008. However, mortality has progressively decreased, with no significant differences observed in the last study period (p = 0.446). After adjusting for age and sex, mortality rates remained similar up to 2008 but were lower among patients with DM in the last study period (OR = 0.89, 95% CI 0.81-0.97, p = 0.012).

Conclusions

In-hospital pneumonia mortality among patients with DM decreased throughout time and was comparable to that of non-DM patients in the final study period.

## Introduction

Pneumonia is among the most important causes of morbidity and mortality worldwide, with a substantial impact on elderly individuals [1,2]. In Portugal, in 2022, respiratory diseases were the third leading cause of mortality, with pneumonia responsible for 3.6% of deaths [3]. Currently, it is also established that diabetes mellitus (DM) is a risk factor for the development of pneumonia, with an adverse effect on the patients’ outcome [4], both due to an increased need for in-patient care and to an independent impact on mortality [5].

A national cross-sectional study in Portugal with patients with type 2 DM showed that in 2019, the in-hospital mortality rate was 9.4%. Among these, the mortality rate from bacterial pneumonia was 30.2% (HR = 3.214; 95% CI = 2.539-4.070) [6].

The literature on the impact of DM on hospital admissions and pneumonia-related mortality in Portugal over the years is scarce [5,6]. Therefore, this study aimed to evaluate the evolution of in-hospital mortality from pneumonia among patients with DM over 25 years at a tertiary care hospital in northern Portugal.

## Materials and methods

Study design and participants

We performed a retrospective cohort study of all adult patients (≥18 years) with or without DM hospitalized at a Portuguese tertiary care hospital between 1989 and 2014 with a primary or secondary diagnosis of community-acquired pneumonia (CAP). Patients were excluded if the diagnosis was coded as hospital-acquired pneumonia or as a noninfectious pulmonary condition. This work has been approved by the Comissão de Ética da Unidade Local de Saúde de São João (approval number: 245-25).

Data collection and definitions

The annual number of hospitalizations for pneumonia, the proportion of hospitalizations for pneumonia, diagnosis of DM, length of stay, in-hospital mortality and demographic data of hospitalized patients (age and sex) were obtained from the hospital’s electronic patient record database (IEG, Virtualcare, Porto, Portugal), which archives diagnostic codes, demographic variables, and hospitalization outcomes in a structured format. Data were retrieved via standardized database queries, and internal consistency checks were performed prior to analysis. CAP diagnosis was defined according to the International Classification of Diseases, 9th Revision, Clinical Modification (ICD-9-CM), which was the version used in the Portuguese health system during this study period. The diagnosis included all ICD-9-CM codes corresponding to CAP (480-486, and 487.0), and the coding was performed by physicians formally trained and certified in clinical codification using the ICD-9-CM, following national standardized procedures. DM was defined according to current diagnostic criteria and based on documented previous medical history of DM, ongoing antidiabetic treatment, or previously documented hyperglycemia (fasting glucose ≥126 mg/dL or hemoglobin A1c ≥6.5% (48 mmol/mol) on at least two separate measurements) in blood glucose evaluation [7].

Statistical analysis

Categorical variables are presented as numbers and percentages. Continuous variables are presented as medians with interquartile ranges due to non-normal distributions. For comparison between patients with and without DM, a chi-square test was performed. In-hospital mortality was evaluated in five periods (years 1989-1993, 1994-1998, 1999-2003, 2004-2008, and 2009-2014) to compare its evolution in patients with and without DM. The 25-year observation period was divided into five 5-year intervals to ensure adequate sample size within each group and to reflect temporal changes in clinical practice and healthcare delivery. Multivariable logistic regression analyses were used to adjust the mortality rate for age and sex. The p-value for trend was calculated using logistic regression with mortality as the outcome and year as a continuous variable. Statistical analyses were performed with Stata® software version 14.1 (StataCorp, College Station, TX, USA). P-values <0.05 were considered significant.

## Results

**Figure 1:**
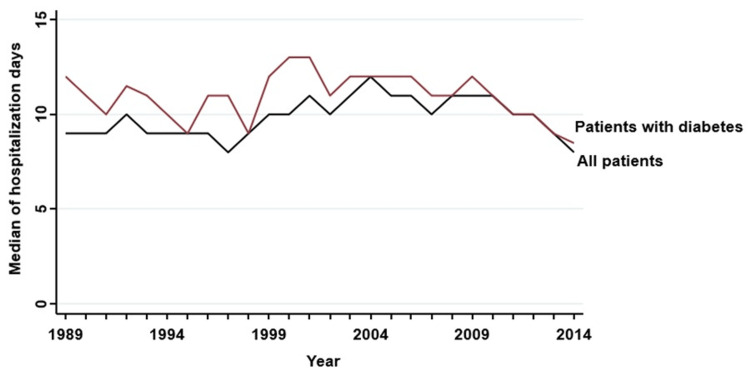
Duration of hospitalizations for pneumonia

During the 25-year study period, 1,356,502 hospitalizations were evaluated (124,524 (9.2%) in patients with DM), of which 44,301 were due to pneumonia (8,991 (20.3%) in patients with DM) (Table 1). The proportion of hospitalizations for pneumonia was higher among patients with DM (7.2% vs 2.9%, p < 0.001). Among patients with DM, the proportion of female patients was higher (46.7% vs 41.3%, p < 0.001). The median age was 68 years (interquartile range 44-79) in the total population and 74 years (66-81) in the population with DM. Patients with a diagnosis of DM had a longer hospital stay than the rest of the population (11 (7-20) vs 10 (6-19) days, respectively) (Figure 1).

**Table 1:** Hospitalizations in a Portuguese tertiary hospital between 1989 and 2014 * Estimated based on data of the total hospitalizations and patients with DM, assuming the same distribution of total hospitalizations. %: percentage, IQR: interquartile range, M: male, F: female, DM: diabetes mellitus

	Hospitalizations	Patients without DM	Patients with DM
Number of hospitalizations, n	1,356,502	1,231,978	124,524
Mortality, %	3.2%	2.9%	6.4%
Number of hospitalizations for pneumonia, n (%)	44,301 (3.3%)	35,310 (2.9%)	8 991 (7.2%)
Sex, %	M 58.2%/F 41.8%	M 58.7%/F 41.3%	M 53.3%/F 46.7%
Age, years (median (IQR))	68 (44-79)	67 (43-78)*	74 (66-81)
Mortality for pneumonia, %	20.2%	19.5%	22.9%
Length of hospitalization, days (median (IQR))	10 (6-19)	10 (6-19)*	11 (7-20)

**Figure 2:**
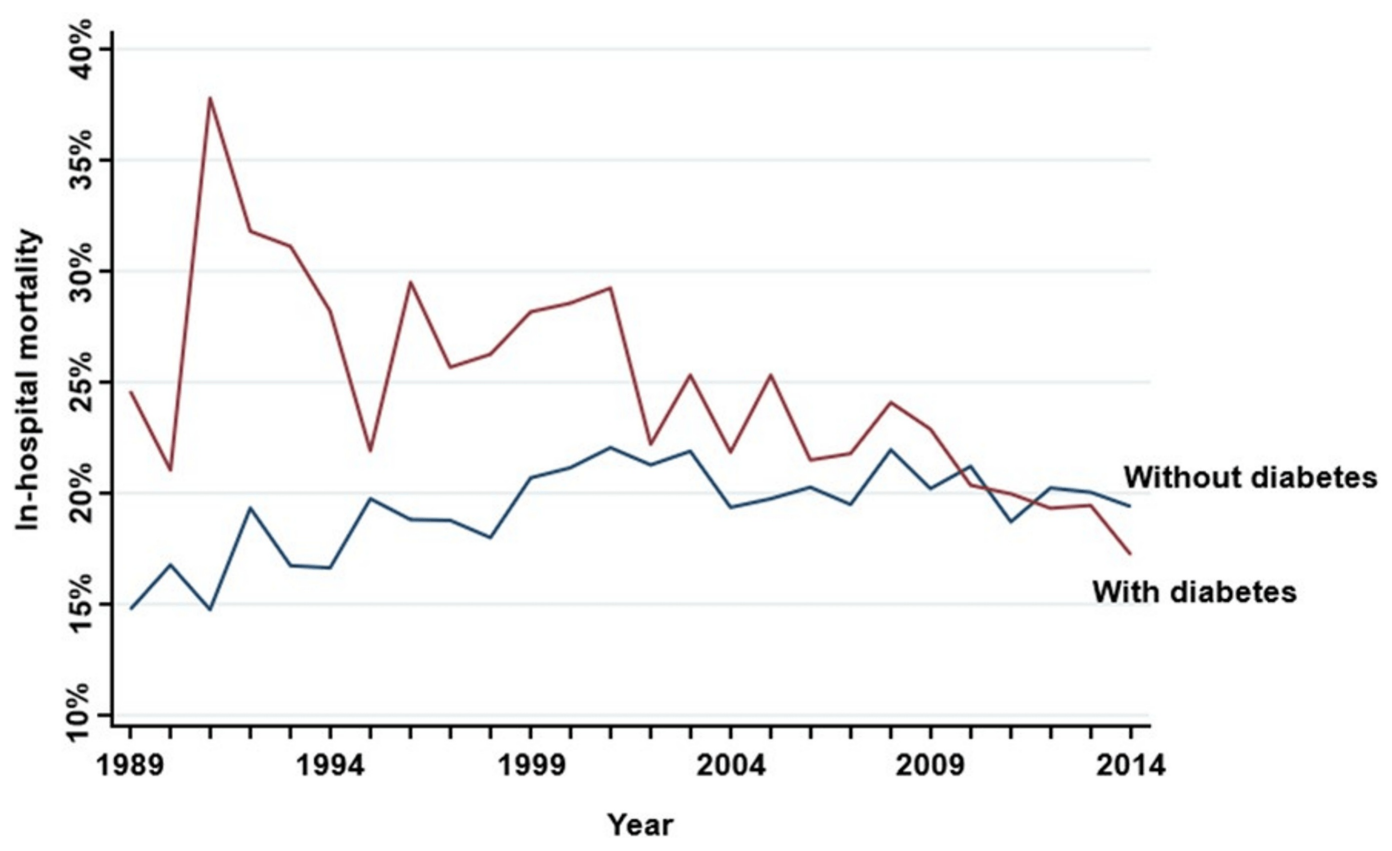
Mortality for pneumonia across time

When compared to patients without DM, patients with DM had a higher in-hospital mortality (p < 0.001) in the various study periods up to the year 2008 (years 1989-1993: 29.8 vs 16.5%; years 1994-1998: 26.2 vs 18.4%; years 1999-2003: 26.3 vs 21.5%; years 2004-2008: 23.0 vs 20.2%) (Table 2). Mortality has progressively decreased in diabetics (Figure 2). In the most recent study interval (2009-2014), there were no significant differences in the in-hospital mortality of patients with and without DM (19.3 vs 20.0%, p = 0.446) (Table 2). After adjusting for age and sex, the in-hospital mortality rate was similar between groups up to 2008. However, during 2009-2014, patients with DM had a lower in-hospital mortality rate (OR = 0.89, 95% CI 0.81-0.97, p = 0.012) (Table 2). The p-for-trend over time was significant in patients with DM (p < 0.001) and in those without DM (p < 0.001).

**Table 2:** Hospitalizations and mortality for pneumonia across time Unadjusted p-values were calculated using chi-square tests comparing proportions between patients with and without DM. * Adjusted p-values and test statistics were obtained from multivariable logistic regression models including age and sex as covariates, using the Wald z test. OR: odds ratio, CI: confidence interval, NA: not applicable, DM: diabetes mellitus

	Patients without DM	Patients with DM	p-value	χ²	Adjusted OR (95% CI)	Adjusted p-value*	Wald z
1989-1993 (n = 210,702)							
Hospitalizations for pneumonia, n (%)	5454 (2.8%)	574 (4.0%)	<0.001	413.7	NA		
Mortality for pneumonia, n (%)	901 (16.5%)	171 (29.8%)	<0.001	74.06	1.19 (0.97-1.46)	0.105	1.62
1994-1998 (n = 226,103)							
Hospitalizations for pneumonia, n (%)	5955 (2.9%)	806 (4.0%)	<0.001	153.5	NA		
Mortality for pneumonia, n (%)	1097 (18.4%)	211 (26.2%)	<0.001	23.15	0.98 (0.82-1.17)	0.791	-0.27
1999-2003 (n = 220,205)							
Hospitalizations for pneumonia, n (%)	6417 (3.2%)	1526 (7.6%)	<0.001	1120	NA		
Mortality for pneumonia, n (%)	1377 (21.5%)	401 (26.3%)	<0.001	21.33	0.96 (0.84-1.10)	0.575	-0.56
2004-2008 (n = 236,744)							
Hospitalizations for pneumonia, n (%)	9014 (4.3%)	2858 (10.1%)	<0.001	1958	NA		
Mortality for pneumonia, n (%)	1825 (20.2%)	656 (23.0%)	0.002	9.39	0.97 (0.88-1.07)	0.530	-0.63
2009-2014 (n = 462,748)							
Hospitalizations for pneumonia, n (%)	8470 (2.0%)	3227 (7.8%)	<0.001	3536	NA		
Mortality for pneumonia, n (%)	1691 (20.0%)	624 (19.3%)	0.446	0.58	0.89 (0.81-0.97)	0.012	-2.51

## Discussion

This is an extensive retrospective analysis of CAP at a Portuguese tertiary center over 25 years. Patients with DM experienced longer hospital stays. Several studies have already demonstrated this [8-10], reinforcing the disease's impact not only on prognosis but also on hospital costs. We point out as contributing factors the greater severity of the disease, the difficulty in managing blood glucose levels during hospitalization, and even the occurrence of hypoglycemia [9-13]. DM is an established risk factor for the development of CAP and other respiratory infections [4] and may be associated with a worse prognosis [5]. Several factors may be accountable for this, namely decreased T-cell-mediated immune response [14], impaired neutrophil function [5], depression of the antioxidant system, or micro- and macroangiopathy [15]. All these pathophysiological mechanisms culminate not only in an increased risk for pneumonia in patients with DM but also in a higher risk of severe disease and death. In our study, we found a higher proportion of hospital admissions for pneumonia among patients with DM than among patients without DM.

Among patients with DM, the proportion of females was higher. This is an unexpected finding, given a higher prevalence of DM in men [16-18] and a higher proportion of men hospitalized with pneumonia in Portugal [19]. However, this can be explained by a longer life expectancy. Most studies agree that life expectancy worldwide, and particularly in Portugal, is higher among women, which supports our argument [20].

Regarding in-hospital mortality, patients with DM had worse outcomes during the first four periods of the study (up to 2008). Interestingly, over time, the mortality gap between patients with and without DM narrowed, and the mortality of patients with DM decreased to the point that, in the last period of the study (2009-2014), there was no difference between the two groups. However, after adjusting for age and sex, there were no differences between groups until the last period, when the mortality rate was lower in the DM group. Anti-pneumococcal vaccination coverage has increased since its introduction in Portugal in 2001 [21], and it is now universally recommended for all patients with DM in national guidelines [22]. This may be responsible not only for a reduction in pneumonia events in immunized patients but also for a reduction in invasive pneumococcal disease, one of the most severe presentations of the disease [21,22]. Influenza vaccination may also play an important role since patients with DM are at higher risk not only for the development of viral pneumonia [23] but also for bacterial superinfection [24]. Advances in DM care, both in long-term care and in the acute management of hyperglycemia during infectious events, may also have contributed significantly to this improvement [25-27]. Finally, progress in medical care, ranging from greater accessibility, improvements in ventilatory and multiorgan support techniques [28], appropriate initial antibiotic selection [29], and other advances that followed national and international guidelines for standard-of-care procedures, was probably no less relevant to this achievement.

Our study has limitations. The data are derived from a database that did not contain direct clinical measures, and we did not have access to clinical records. As a result, our multivariable models could adjust for age and sex but not for other potential confounding factors, namely those related to DM and CAP severity, therapies and vaccination, smoking history, lifestyle, or other comorbidities. Therefore, the mortality differences observed should be interpreted as descriptive rather than causal. Additionally, changes over the 25 years under analysis, including variations in admission policies, clinical practice patterns, diagnostic criteria, and coding practices, may introduce bias in a long-term retrospective study such as this one. Despite these limitations, the sample size and period of observation help mitigate them, allowing a robust description of long-term trends in pneumonia hospitalizations among patients with and without DM. The sample size and extensive observation period are the study’s main strengths.

## Conclusions

In-hospital mortality from pneumonia in patients with DM has been declining throughout the years. By the final interval in our study, it was comparable to that of patients without DM. Several improvements in DM and pneumonia care occurred in Portugal during this timeframe, including increasing pneumococcal and influenza vaccination coverage. However, these factors were not evaluated in this study. Therefore, no causal inferences can be made, and the observed trends should be interpreted as descriptive findings. Further prospective studies are needed to determine causality.
